# Clinical Presentation and Treatment Outcomes of Children and Adolescents With Pheochromocytoma and Paraganglioma in a Single Center in Korea

**DOI:** 10.3389/fendo.2020.610746

**Published:** 2021-01-29

**Authors:** Hyojung Park, Min-Sun Kim, Jiwon Lee, Jung-Han Kim, Byong Chang Jeong, Sanghoon Lee, Suk-Koo Lee, Sung Yoon Cho, Dong-Kyu Jin

**Affiliations:** ^1^Department of Pediatrics, Samsung Medical Center, Sungkyunkwan University School of Medicine, Seoul, South Korea; ^2^Department of Pediatric Surgery, Samsung Medical Center, Sungkyunkwan University School of Medicine, Seoul, South Korea; ^3^Department of Urology, Samsung Medical Center, Sungkyunkwan University School of Medicine, Seoul, South Korea

**Keywords:** pheochromocytoma, paraganglioma, adrenal mass, hypertension, pediatrics

## Abstract

**Purpose:**

Pheochromocytoma (PCC) and paraganglioma (PGL) (PPGL) are rare neuroendocrine tumors, and data on managing these conditions in children and adolescents are lacking. The objective of this study was to demonstrate the clinical presentation and treatment outcomes in children and adolescents with PPGL in a single tertiary care center in Korea.

**Methods:**

This retrospective study included 23 patients diagnosed with PCC (*n* = 14) and PGL (*n* = 9) before the age of 21 at Samsung Medical Center (from June 1994 to June 2019). We describe age, gender, family history, clinical characteristics, laboratory findings, pathologic findings, therapeutic approaches, and treatment outcomes.

**Results:**

Of the 23 patients, 14 had PCC and nine had PGL. The median age at diagnosis was 16.8 years (range, 6.8–20.8 years). The common presenting symptoms were hypertension (*n* = 10), headache (*n* = 9), palpitation (*n* = 4), and sweating (*n* = 4). The plasma or 24-hour urine catecholamine and/or metabolite concentrations were markedly elevated in 22 patients with PPGL, but were normal in one patient with carotid body PGL. All tumors were visualized on computed tomography. Genetic tests were performed in 15 patients, and seven patients showed mutations in *RET* (*n* = 3), *SDHB* (*n* = 3), and *VHL* (*n* = 1). All patients underwent surgery, and complete excision was performed successfully. Three patients with metastasis underwent postoperative adjuvant therapy.

**Conclusion:**

This study suggests that pediatric PPGL tends to be extra-adrenal and bilateral and shows a higher potential for genetic mutations. Considering the hereditary predisposition of pediatric PPGL, genetic screening tests are strongly recommended, and lifelong follow-up is needed to detect recurrence and metastasis. Further research with a larger sample size and routine genetic screening is needed to better understand the genetic conditions and long-term prognosis of PPGL.

## Introduction

Pheochromocytoma (PCC) and paraganglioma (PGL) (PPGL) are rare neoplasm, which arise from chromaffin cells that release catecholamines, including epinephrine (EPI), norepinephrine (NE), and dopamine (1). PCCs are located in the adrenal medulla, whereas PGLs arise in extra-adrenal locations, such as the sympathetic/parasympathetic ganglia (1, 2). Although the incidence of PPGL in the pediatric population is 0.2–0.5 cases per million, they are the most common endocrine tumors in childhood and account for 0.5–1% of pediatric hypertensive cases (3–5). The average age at diagnosis is 11–13 years for pediatric PPGL, with the incidence rate in males being twice that of the rate in females (1). The symptom triad of PPGL includes headache, sweating, and palpitation (6). In children, persistent hypertension is the most frequent symptom, unlike the paroxysmal hypertension observed in adults (1, 6, 7). Other symptoms of PPGL include anxiety, weight loss, visual disturbance, polyuria, and polydipsia (1, 6, 7). In adults, the “rule of 10%” for PPGLs implies that 10% are malignant, 10% are bilateral, 10% are extra-adrenal, 10% are hereditary, and 10% are in children (8). With advancements in diagnostic techniques and genetic testing, the prevalence of bilateral, extra-adrenal, hereditary, and metastatic PPGLs has changed since the original “rule of 10%” for PPGL (9). It has been reported that 50–70% of pediatric PPGL cases are associated with germline mutations in known susceptibility genes (10). The standard treatment for PPGL is total adrenalectomy (11). Unlike other tumors, there are no clinical, biochemical, or histopathological criteria for malignant PPGL (12, 13). The 2017 World Health Organization (WHO) classification has defined malignant PPGL as a metastatic disease that is histologically diagnosed by the presence of chromaffin tissues in organs that usually lack chromaffin cells (14). However, data for pediatric PPGL are limited, and the clinical management of PPGL in children and adolescents is usually extrapolated from adult data (8). Hence, the objective of this study was to demonstrate the clinical presentation and treatment outcomes of children and adolescents with PPGL in a single tertiary care center in Korea.

## Patients and Methods

This study included 23 patients diagnosed with PCC (*n* = 14) and PGL (*n* = 9) before the age of 21 at Samsung Medical Center (June 1994 to June 2019). Diagnosis was based on histopathologic findings from surgical specimens. Data on age at diagnosis, gender, family history, clinical characteristics, laboratory findings, pathologic findings, therapeutic approaches, and treatment outcomes were collected retrospectively based on medical records. This study was approved by the Institutional Review Board of Samsung Medical Center (2020–03–202).

The concentrations of metanephrine (MN), normetanephrine (NMN), EPI, and NE in 24-hour urine or plasma were measured. Patients underwent computed tomography (CT) and/or magnetic resonance imaging (MRI) of the abdomen. Specific functional imaging with radioactive meta-iodobenzylguanidine (MIBG) scintigraphy or fluorodeoxyglucose-positron emission tomography (PET) was performed to describe metastasis and biochemically silent disease. Genetic tests for multiple endocrine neoplasia syndrome (MEN)-2A and 2B (*RET*), neurofibromatosis type I (*NF1*), Von Hippel Lindau (*VHL*) syndrome, and PGL with succinate dehydrogenase complex subunit B (*SDHB*), D (*SDHD*), and C (*SDHC*) were performed to identify hereditary diseases. In the revised WHO classification, there is no specific histological system to evaluate biological aggressiveness and thus, malignant PPGL was defined as a metastatic disease.

## Results

### Clinical Presentation and Diagnostic Evaluations of Patients With PPGL

Of the 23 study patients, 14 patients (10 male and four female patients) had PCCs, and nine patients (five male and four female patients) had PGLs. The median age at diagnosis was 16.8 years (range, 6.8–20.8 years). Clinical presentations and diagnostic evaluations of the 23 patients with PPGL are shown in **Table 1**. The most common presenting symptoms were hypertension (*n* = 10), headache (*n* = 9), palpitation (*n* = 4), and sweating (*n* = 4), listed in the order of frequency. Three patients were incidentally diagnosed with PPGL during general health screening. The plasma or 24-hour urine catecholamine and/or metabolite concentrations were markedly elevated in 22 patients with PPGL, but were normal in one patient with carotid body PGL. The 24-hour urine MN or EPI concentrations were mainly elevated in PCC, but not PGL. Similarly, the plasma NMN or NE concentrations were higher in PGL cases than in PCC cases (**Figure S1**). After surgery, the concentrations of catecholamines and their metabolites normalized in all patients. CT was the most commonly utilized diagnostic tool in our study. All tumors were visualized on CT. To detect metastatic or multifocal disease, functional imaging with MIBG scintigraphy or PET was performed in conjunction with CT or MRI. In the PCC group, five patients had a tumor on the bilateral adrenal gland and nine patients had a unilateral mass. Most PGLs occurred along the sympathetic chain in the retroperitoneum. Only one patient with PGL had a tumor in the carotid bifurcation of the neck. The median maximal diameter of the tumors was 4.75 cm (range, 1.5–19.0 cm).

**Table 1:** Clinical presentations and diagnostic evaluation of patients with PPGL.

Age at diagnosis, median (range) years	Total (23)		PCC (14)		PGL (9)	
	16.8 (6.8–20.8)		17.6 (13.3–20.3)		13.7 (6.8–20.8)	
**Gender, n (%)**	M	F	M	F	M	F
	15 (65.2%)	8 (34.8%)	10 (71.4%)	4 (28.6%)	5 (55.6%)	4 (44.4%)
**Main presenting symptoms**						
Hypertension Headache Palpitation Sweating Incidental	10 9 4 4 2		7 5 4 3 1		3 4 0 1 1	
**Catecholamine and/or metabolite in plasma or 24-hour**						
**urine, n (%)**						
Elevated Normal	22 (95.7%) 1 (4.3%)		14 (100%) 0		8 (88.9%) 1 (11.1%)	
**Imaging performed to diagnose**						
CT MRI MIBG PET	22 2 8 9		13 0 4 8		9 2 4 1	
**Location of primary tumor, n (%)**			Unilateral 9 (64.3%) Bilateral 5 (35.7%)		Neck 1 (11.1%) Retroperitoneum 8 (88.9%)	
**Metastasis**	3 (13%)		1 (7.1%)		2 (22.2%)	
**Recurrence**	3 (13%)		3 (21.4%)		0 (0%)	

PPGL, PCC and PGL; PCC, pheochromocytoma; PGL, paraganglioma; CT, computed tomography; MRI, magnetic resonance imaging; MIBG, meta-iodobenzylguanidine scintigraphy; PET, positron emission tomography; M, male; F, female.

### Genetic Evaluation of PPGL

Genetic tests were performed in 15 patients, and mutations related to PPGL were identified in seven patients (**Table 2**). Among patients with PCCs, three had an *RET* mutation and one had a *VHL* mutation. Among patients with PGL, three had an *SDHB* mutation. Two patients with an *RET* mutation had bilateral PCC and underwent total thyroidectomy because of medullary thyroid carcinoma. The other patient with an *RET* mutation had unilateral PCC, autoimmune thyroiditis, and type 1 diabetes mellitus. The patient with a *VHL* mutation had cerebellar hemangioblastoma 6 years after bilateral adrenalectomy. Two of the three PGL patients with *SDHB* mutations had metastasis identified at diagnosis or during follow-up and received adjuvant therapy. The other patient, who was diagnosed with PGL and had an *SDHB* mutation, did not show other abnormalities, such as metastasis, during follow-up.

**Table 2:** Clinical characteristics of patients with PPGL.

Patients	Sex	Age at diagnosis(y)	Initial presentation	BP at diagnosis(mmHg)	Tumorlocation	Maximal tumor diameter (cm)	Surgical approach	Post-operative treatment	Follow-up duration (y)	Recurrence	Metastasis	Family history	Co-morbidity	Mutation
**PCC1**	M	19.3	Incidental	146/87	Lt	4.5	Lapa	N	3.3^#^	N	N	N	N	ND
**PCC2**	M	13.3	HTN	135/79	BL	Rt 4.5 Lt 4.5	Open	Y	Transfer	Y	N	N	N	NA
**PCC3**	M	16.4	HTN	158/76	BL	Rt 4 Lt 3	Open	Y	11.4	N	N	N	Cerebellar HB	*VHL*
**PCC4**	F	18.3	HTN	145/99	Lt	5	Open	N	9.8	N	N	N	N	NA
**PCC5**	M	13.6	HA	115/68	BL	Rt 8 Lt 5	Open	Y	7	N	N	N	DCMP, Hypothyroidism	ND
**PCC6**	M	18.5	Incidental	129/69	Lt	3	Lapa	N	3.1	N	N	N	N	ND
**PCC7**	F	13.5	HA	165/125	Lt	4.3	Open	N	2.7	N	N	N	N	ND
**PCC8**	F	16.5	Palpitation	160/110	Lt	10.5	Open	N	2.3	N	N	N	N	ND
**PCC9**	M	20.2	HTN	155/101	BL	Lt 6.5 Rt 1.8	Lapa	Y	7.9	Y	N	N	MTC	*RET*
**PCC10**	M	20.3	Palpitation	102/63	Lt	9	Open	N	4	N	N	N	N	ND
**PCC11**	M	20	HTN	133/74	Rt	7.5	Open	CT	13.5	N	Y (synchronous)	N	N	NA
**PCC12**	M	16.8	HTN	160/110	Lt	7.5	Open	N	12.8^#^	N	N	N	N	NA
PCC13^*^	F	19.9	HA	115/56	BL	Rt 3.8 Lt 0.9	Open	Y	14.1^#^	Y	N	Y	N	*RET*
**PCC14**	M	16.9	Vomiting	140/88	Rt	6.7	Lapa	N	0.8	N	N	N	Autoimmune thyroiditis DM	*RET*
**PGL15**	M	18.3	Dyspnea	145/75	RP	7	Open	Y	0.7^#^	N	N	N	N	NA
**PGL16**	M	13.7	HTN	Systolic BP>160	RP	3	Open	N	0.6^#^	N	N	N	N	NA
**PGL 17**	F	15.5	HTN	130/85	RP	4.5	Open	N	1.5^#^	N	N	N	N	NA
**PGL18**	M	20.7	Abdominal mass	127/75	RP	19	Open	CT, RT, MIBG	19.9	N	Y (metachronous)	N	N	*SDHB*
**PGL19**	F	9.3	HTN	160/110	RP	6.5	Open	N	10.3	N	N	N	DCMP	ND
**PGL20**	F	7.3	Abdominal mass	121/60	RP	9.5	Open	CT, MIBG	3	N	Y (synchronous)	N	N	*SDHB*
**PGL21**	M	20.8	Neck mass	122/60	Carotid	5	Open	N	0.5^#^	N	N	N	N	ND
**PGL22**	M	12.8	Incidental	106/50	RP	4.3	Open	N	0.5^#^	N	N	N	N	*SDHB*
**PGL23**	F	6.8	HTN	160/110	RP	1.5	Open	N	3.1	N	N	N	N	NA

PPGL, PCC and PGL; PCC, pheochromocytoma; PGL, paraganglioma; M, male; F, female; y, years; HTN, hypertension; HA, headache; BP, blood pressure; Lt, left; BL, bilateral; Rt, right; RP, retroperitoneum; Lapa, laparoscopic; Y, yes; N, no; CT, chemotherapy; RT, radiotherapy; MIBG, meta-iodobenzylguanidine scintigraphy; HB, hemangioblastoma; DCMP, dilated cardiomyopathy; MTC, medullary thyroid carcinoma; DM, diabetes mellitus; ND, not detected; NA, not available; VHL, Von Hippel Landau; SDHB, succinate dehydrogenase complex subunit B.

* : positive family history (mother and uncle) ^#^: this information is based on the active monitoring period.

### Therapeutic Approaches and Treatment Outcomes

All patients underwent surgery and complete excision successfully. In our cohort, all patients started to take an alpha-blocker 1 to 2 weeks before surgery to prevent hypertensive crisis and hypotensive shock. A beta-blocker was added to control tachyarrhythmia or reflex tachycardia in eight patients. As a result, hypertensive crisis, stroke, arrhythmia, or myocardial infarction did not occur during or after surgery. Nineteen patients underwent laparotomy, and four patients underwent laparoscopic surgery. All patients had negative microscopic margins in their pathological samples. Capsular invasion was identified in four patients with PCC and in two patients with PGL. Vascular invasion was identified in one patient with PCC and one with PGL. Lymphatic invasion was identified in only one PGL patient. There was no mortality related to surgery, postoperative hypotension, or hypoglycemia. One patient had renal atrophy due to left renal vein thrombosis. The median follow-up period was 39 months (range, 6 months to 19.9 years). Three patients with PCC showed bilateral metachronous lesions, and the time to recurrence was 7, 42, and 51 months in these three patients. In patients with PGL, no recurrence occurred during the follow-up period. One of the 14 patients with PCC and two of the nine patients with PGL were classified as having metastasis. The sites of metastasis were mainly the lungs, bones, and lymph nodes. One patient with PGL showed metachronous metastasis after 8 years and 11 months from tumorectomy. The other patient with PGL and one patient with PCC had synchronous metastasis at diagnosis. These three patients with metastasis underwent adjuvant therapy. One patient with PCC was treated with chemotherapy and transferred to another hospital. One patient with PGL is currently receiving [^131^I] MIBG treatment followed by chemotherapy. The other with PGL received chemotherapy, radiotherapy, and MIBG treatment. No patient died during the follow-up period. Therapeutic approaches and treatment outcomes are summarized in **Table 3**.

**Table 3:** Therapeutic approaches and treatment outcomes.

	Total	PCC	PGL
**Surgical treatment, n (%)**			
Laparoscopic	4 (17.4%)	4 (28.6%)	0
Open	19 (82.6%)	10 (71.4%)	9 (100%)
**Chemotherapy, n (%)**	3 (13%)	1 (7.1%)	2 (22.2%)
**Radiotherapy, n (%)**	1 (4.3%)	0	1 (11.1%)
**MIBG, n (%)**	2 (8.7%)	0	2 (22.2%)

PCC, pheochromocytoma; PGL, paraganglioma; MIBG, meta-iodobenzylguanidine scintigraphy.

## Discussion

This study describes the clinical presentation and treatment outcomes of PPGL in children and adolescents.

Owing to the rarity of PPGL, specific guidelines have not been adequately described for the pediatric population in the literature. In this population, the presentation of PPGL ranges from ambiguous symptoms to hypertensive crisis.

Symptomatology depends on the type of catecholamines secreted by PPGL (15, 16). The surge of catecholamines may be continuous or intermittent from the PPGL, resulting in various manifestations. Patients with NE-secreting tumors generally show persistent hypertension, whereas those with EPI-secreting tumors exhibit paroxysmal hypertension (15–17). However, PPGL can also present with nonspecific symptoms or an incidental mass. A retroperitoneal mass in children is likely to be mistaken for Wilms’ tumor or neuroblastoma rather than PPGL. Thus, the diagnosis of PPGL is based on the clinical presentation and the elevation of catecholamines or their metabolites in plasma or urine (6). The secretion of EPI is usually limited to PCC rather than PGL because PGL predominantly secretes NE. As PCCs can secrete both NE and EPI owing to the phenylethanol-*N*-methyltransferase enzyme, concentrations of EPI and its metabolite, MN, are elevated. As NE is mainly secreted in PGLs, concentrations of MN and EPI are not elevated (4, 17, 18). The small number of patients with PCC and PGL was a drawback in the determination of statistically significant differences in biochemical profiles between PCC and PGL. In our patients with PGL, the concentrations of EPI and MN in plasma or 24-hour urine were not as high as those in patients with PCC (**Figure S1**). In our study, plasma or 24-hour urine catecholamine and/or metabolite concentrations were markedly elevated in 22 patients with PPGL, but were normal in one patient with carotid body PGL. Carotid body PGL presents with a bulging neck mass and is a nonsecreting tumor. Head and neck PGLs from parasympathetic tissue are mostly chromaffin-negative tumors, and only 4% of parasympathetic PGLs secrete catecholamines (19). Recently, measurement of plasma or urine MN and NMN concentrations has been recommended as the most sensitive test for PPGL diagnosis (1, 4). CT or MRI has been recommended owing to their similar sensitivity in localizing pediatric PPGL (90–100%) (1, 18, 20). In our study, all tumors were visualized on CT. MRI is recommended especially in patients with metastatic PPGL, patients with surgical clips, and in pediatric patients to avoid radiation exposure. MRI is superior to CT for identifying the vascular structures of PCC (1). More recently, functional imaging with MIBG and PET has been complemented with CT or MRI to detect metastasis and recurrence and to evaluate tumor response to adjuvant therapy (1, 18, 20).

Up to 70–80% of pediatric patients with PPGL have an associated germline mutation, which may or may not be hereditary in nature or associated with a genetic syndrome (21). Approximately 40% of pediatric patients with PPGL have a hereditary disposition, and *MEN2, VHL, NF1*, and *SDHx* constitute the best-known genetic syndromes associated with PPGL. Considering this strong association, genetic testing is recommended in all children and adolescents with PPGL (22, 23). *VHL* is the major gene in children with PCC, and *SDHB* is associated with PGL. In particular, *SDHx* genes are associated with metastatic potential in PPGL (24).

Fifteen patients (65.2%) underwent genetic testing for mutations in PPGL-susceptibility genes; a pathogenic mutation was found in seven of them (46.7%). Because this retrospective study covers a long period, genetic screening was not proposed as a mandatory test for all patients. We identified known pathogenic mutations in *VHL*, *RET*, and *SDHB* in seven patients. A new analysis by next-generation sequencing with a panel including all identified PPGL-susceptibility genes should be considered to identify a pathogenic mutation in patients with previous negative genetic testing results.

Preoperative antihypertensive preparation is essential in PPGL surgery to avoid blood pressure fluctuations or a hypertensive crisis (1, 16). In children, phenoxybenzamine is widely recommended as an alpha-blocker. A high sodium diet and fluids totaling 1.5 times maintenance are required to maintain intravascular volume before surgery (1).

The definite treatment of PPGL is surgical resection. The surgical approach for a PPGL should be planned according to the secretion status of the tumor, the presence of bilateral lesions, and the surgeon’s experience. In the past, laparotomy was the main surgical procedure in our center to minimize risk of recurrence and metastasis based on the surgeon’s judgement. Recently, minimally invasive laparoscopic excision has been recommended in PPGL if there is no finding of multiple lesions, infiltration, metastasis, or vascular invasion around the tissue (4, 9, 25, 26). Cortical-sparing surgery is preferred in children to avoid lifelong glucocorticoid and mineralocorticoid replacement after bilateral adrenalectomy (4, 25–27). In our study, all patients showed a resectable lesion and underwent surgical resection successfully. No patients underwent adrenocortical-sparing surgery.

Systemic cytotoxic chemotherapy with cyclophosphamide, vincristine, and dacarbazine is indicated in patients with unresectable and rapidly progressive metastatic PPGL (26). In our study, although three patients who received chemotherapy showed mild anorexia and neutropenic fever, they did not show severe adverse effects such as catecholamine crisis. All patients underwent adrenalectomy in our study, and the proportion of patients with synchronous metastasis at diagnosis was lower (*n*=2) than that in other studies (28, 29). The higher survival rate compared to that in other studies (100 vs 72–97%) seems to be attributed to these factors (6, 10). However, long-term monitoring should be performed because late events could still occur.

Two patients (PCC 5 and PGL 19) were diagnosed with dilated cardiomyopathy. Excessive catecholamine can stimulate cardiac myocytes and lead to myopathy. Both patients presented with headache and hypertension. After surgery, their blood pressure was stabilized and remained within normal limits without antihypertensive drugs. Their cardiac function improved, and they did not have any features of cardiac failure thereafter.

Advancements in diagnostic techniques and genetic testing have broken the “rule of 10%” in PPGL. In recent years, more than 50% of pediatric PPGL have been reported with germline mutations in known susceptibility genes (10). As reported in several studies, pediatric PPGL is characterized by extra-adrenal, bilateral, and various phenotypes, indicating the differences in disease presentation in children and adults. In a large study, children showed higher prevalence of hereditary (80.4 vs. 52.6%), extra-adrenal (66.3 vs. 35.1%), multifocal (32.6 vs. 13.5%), metastasis (49.5 vs. 29.1%), and recurrent (29.5 vs. 14.2%) PPGLs than did adults (29). In another review of pediatric PPGL, 10% were metastatic, 20% were synchronous bilateral, 30% were extra-adrenal, and 40% were familial (28). Our study showed comparable results. First, the proportion of extra-adrenal was 39.1% (9/23) in our study. Second, the incidence of bilateral PCC was 35.7% (5/14). Third, 60% (3/5) of patients with PGL who had undergone genetic testing were identified as having the *SDHB* mutation, while 40% (4/10) of patients with PCC were confirmed with genetic mutations. Lastly, the rate of metastasis in our study was 7.1% (1/14) for PCC and 22.2% (2/9) for PGL. These results are consistent with the fact that PGL has a greater metastatic potential (12).

This study has several limitations. As PPGL is rare in children and adolescents, the size of the study population was insufficient for statistical analysis. Routine genetic testing could not be conducted consistently in all patients. For these reasons, the detection rate of genetic mutations may be inaccurate. We need to perform genetic tests for patients who have not had this testing done in the past. Moreover, a new analysis by next-generation sequencing with a panel including all identified PPGL-susceptibility genes can also identify a pathogenic mutation in patients with previous negative genetic testing results. Lastly, as this study examined rare, pediatric neuroendocrine diseases in a single large center, a multicenter study is needed to obtain more information.

## Conclusion

PPGLs are rare neuroendocrine tumors, and experience managing pediatric PPGLs is not extensive. This study suggests that pediatric PPGL tends to be extra-adrenal and bilateral and shows a higher potential for genetic mutations. Considering the hereditary predisposition of pediatric PPGL, genetic screening tests are strongly recommended, and lifelong follow-up is needed to detect recurrence and metastasis. Further research with a larger sample size and routine genetic screening is needed to better understand the genetic conditions and long-term prognosis of PPGL.

## Data Availability Statement

The original contributions presented in the study are included in the article/supplementary material. Further inquiries can be directed to the corresponding authors.

## Ethics Statement

Written informed consent was obtained from patients and the parents of each patient. The Institutional Review Board at Samsung Medical Center approved this study (IRB file number: 2020-03-202).

## Author Contributions

HP, SC, and D-KJ contributed to the conception and design of the study. HP and M-SK organized the database. M-SK and JL performed the manual segmentation. HP, M-SK, and JL performed the statistical analysis. HP wrote the first draft of the manuscript. SC, SL, S-KL, and J-HK wrote sections of the manuscript. All authors contributed to the article and approved the submitted version.

## Funding

This study was supported by a grant from Samsung Medical Center (GFO3200061).

## Conflict of Interest

The authors declare that the research was conducted in the absence of any commercial or financial relationships that could be construed as a potential conflict of interest.

## Supplementary Material

Supplementary Figure 1 Plotting of biochemical values in patients with PPGL. Unfilled and filled squares represent biochemical values in PCC within and out of normal range, respectively. Unfilled and filled circles represent biochemical values in PGL within and out of normal range, respectively. Normal ranges of each biochemical value were obtained from the Tietz textbook of Clinical Chemistry & Molecular Diagnostics, 5th ed (30). PPGL, PCC and PGL; PCC, pheochromocytoma; PGL, paraganglioma; NMN, normetanephrine; MN, metanephrine; NE, norepinephrine; EPI, epinephrine.
