# Supplementary figures and images for: Clinical Presentation and Treatment Outcomes of Children and Adolescents With Pheochromocytoma and Paraganglioma in a Single Center in Korea
Source: Front Endocrinol (Lausanne). 2021 Jan 29;11:610746. doi: 10.3389/fendo.2020.610746 (PMC7879705; doi:10.3389/fendo.2020.610746)

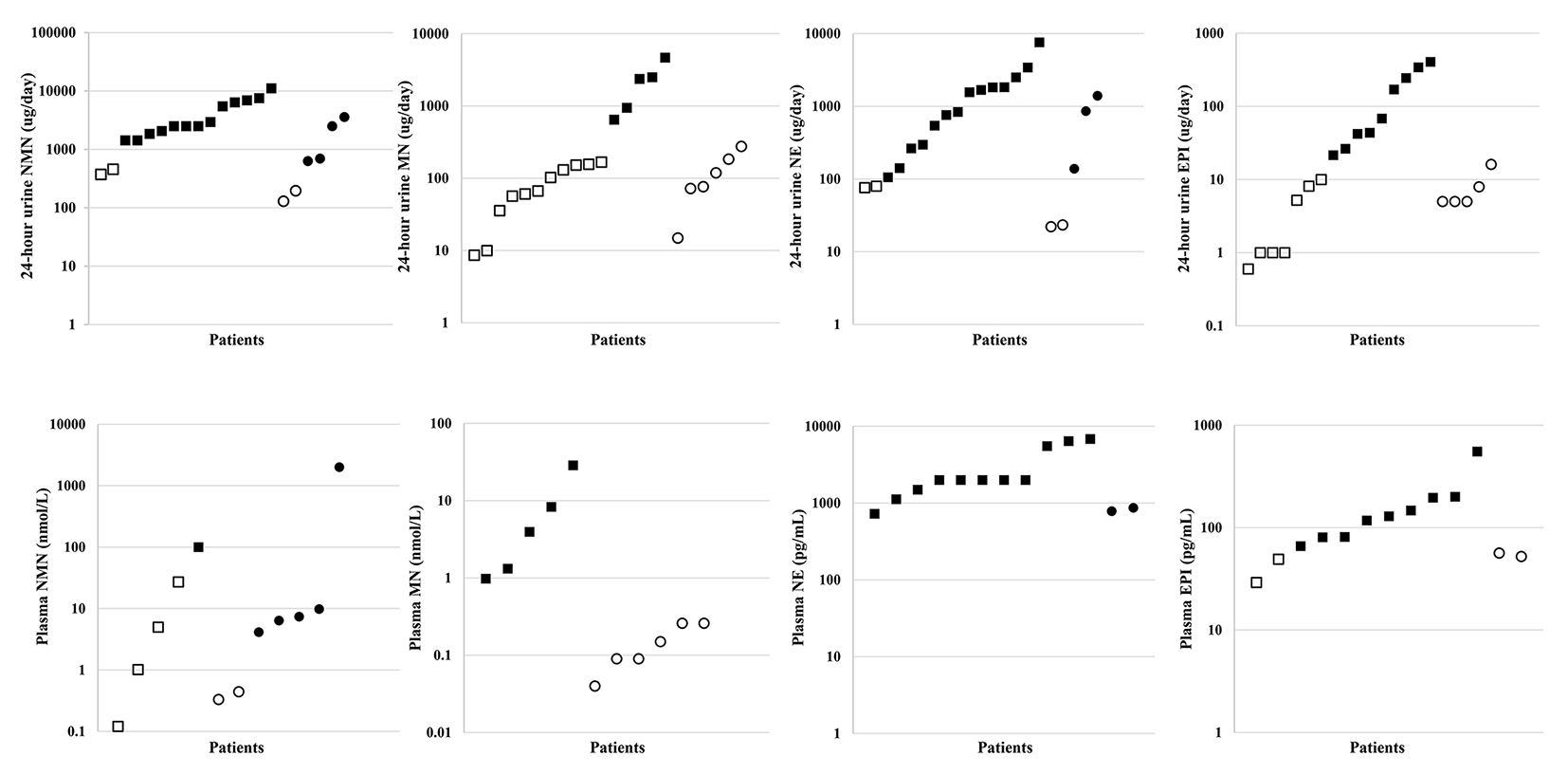

Supplement: Supplementary Figure 1 — Plotting of biochemical values in patients with PPGL. Unfilled and filled squares represent biochemical values in PCC within and out of normal range, respectively. Unfilled and filled circles represent biochemical values in PGL within and out of normal range, respectively. Normal ranges of each biochemical value were obtained from the Tietz textbook of Clinical Chemistry & Molecular Diagnostics, 5th ed (30). PPGL, PCC and PGL; PCC, pheochromocytoma; PGL, paraganglioma; NMN, normetanephrine; MN, metanephrine; NE, norepinephrine; EPI, epinephrine. [file Image_1.tif]
